# Beyond Allotypes: The Influence of Allelic Diversity in Antibody Constant Domains

**DOI:** 10.3389/fimmu.2020.02016

**Published:** 2020-08-18

**Authors:** Annmaree K. Warrender, William Kelton

**Affiliations:** Te Huataki Waiora School of Health, The University of Waikato, Hamilton, New Zealand

**Keywords:** allotypes, Fc receptor, antibody constant domain, antibody alleles, antibody polymorphism, antibody effector functions, heavy chain diversity, immunoglobulin diversity

## Abstract

Polymorphic diversity in antibody constant domains has long been defined by allotypic motifs that cross react with the sera of other individuals. Improvements in sequencing technologies have led to the discovery of a large number of new allelic sequences that underlie this diversity. Many of the point mutations lie outside traditional allotypic motifs suggesting they do not elicit immunogenic responses. As antibodies play an important role in immune defense and biotechnology, understanding how this newly resolved diversity influences the function of antibodies is important. This review investigates the current known diversity of antibody alleles at a protein level for each antibody isotype as well as the kappa and lambda light chains. We focus on evidence emerging for how these mutations perturb antibody interactions with antigens and Fc receptors that are critical for function, as well as the influence this might have on the use of antibodies as therapeutics and reagents.

## Introduction

Antibodies play an essential role as frontline molecules of adaptive immunity in the fight against infection and disease. While pathogenic targets can be directly neutralized via binding of antibody variable domains, binding of the constant domain to specialized Fc receptors on the surface of immune cells dictates powerful inflammatory or anti-inflammatory responses (1). Together the remarkable affinity, exquisite specificity, and immune modulation potency of these interactions have seen antibodies adopted as indispensable reagents in medicine and as diagnostics.

The profile of Fc receptor engagement is, in part, governed by the heavy chain isotype of the antibody encountered. Immunoglobulin (Ig) isotypes are divided into classes and subclasses depending on the heavy chain they possess; IgM (μ), IgD (δ), IgG (γ) which encompasses subclasses IgG1, IgG2, IgG3, IgG4, IgA (α) which encompasses subclasses IgA1 and IgA2, and IgE (ε) (2). Each isotype is paired with either a kappa (κ) or lambda (λ) light chain to create a tetrameric immunoglobulin complex capable of triggering unique effector functions. Adding a further level of complexity, and in spite of the “constant” naming convention, genes of the immunoglobulin heavy-chain constant (IGHC) and light-chain constant (IGLC) loci are polymorphic, although to a far lesser extent than the immunoglobulin heavy-chain variable locus (3). IGHC/IGLC polymorphic diversity has historically been defined by amino-acid mutations in the polypeptide chain that are immunogenic. Termed allotypes, these mutations were first identified by *ex vivo* serological studies in which antibodies from donor sera were observed to trigger agglutination of erythrocytes treated with host serum (4, 5). Allotypes have been identified for IgG1, IgG2, IgG3, IgG4, IgA2, and IgE as well as the kappa light chain (6–8). Before the ready availability of genetic sequencing techniques, allotyping provided an excellent method to track genetic linkage between human populations and ethnic groups thus allowing deeper understanding of human migration patterns (9), population genetics (10), and created tools in forensic medicine (11).

More recently, allotypes have been investigated for their potential role as immunogenic motifs in therapeutic antibodies. Surprisingly, it appears that allotypes act only as minor epitopes in monoclonal antibodies and do not appear to elicit acute rejection (12), although some studies have reported low levels of pre-existing circulating antibodies against allotypes of therapeutic monoclonals (13, 14). A key pitfall of serological detection of allotypes is the difficulty of evaluating immunogenic responses to immunoglobulin isotypes expressed at low levels in serum, such as IgE and IgM, signified by a lack of known allotypes for these classes (8). Furthermore, serological reagents used for allotype detection are of limited availability, and sourcing of anti-sera for rare motifs is difficult, meaning some motifs may be missed (10, 15).

**FIGURE 1:**
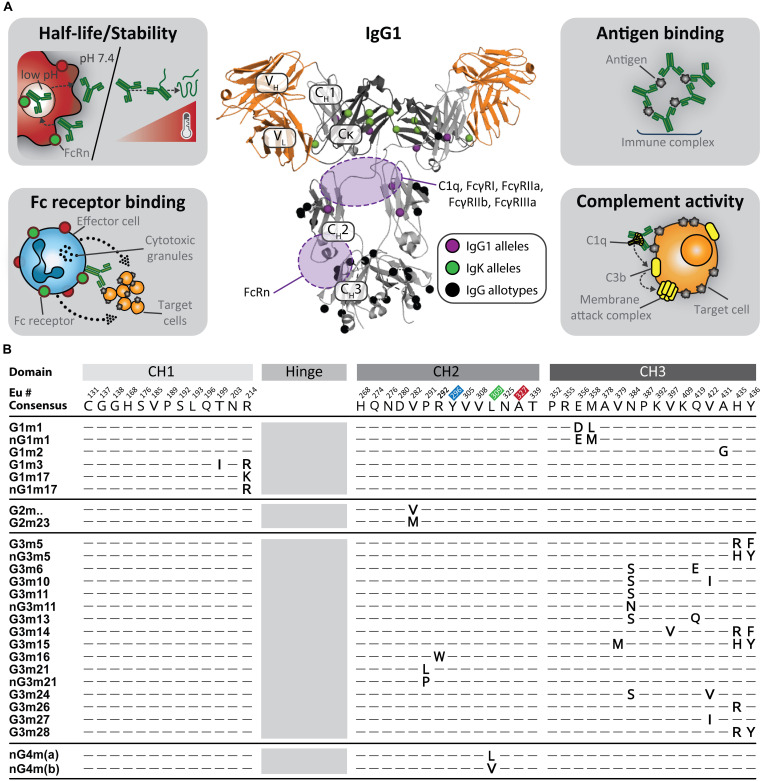
**(A)** The influence of antibody constant domain diversity on function. Amino acid differences in antibody alleles perturb interaction with Fc receptors, antigens, and complement proteins, as well as influencing intrinsic antibody stability. Shown in the center is a crystal structure of full length IgG1 [PDB: 1hzh (80)] with mapped allotype locations (black), additional IgG1 point mutations coded by alleles (purple), and IgK point mutations coded by alleles (green). The light chain comprises the V_*L*_ and Cκ domains whereas the heavy chain is made of the V_*H*_, C_*H*_1, C_*H*_2, and C_*H*_3 domains. Orange indicates the domains responsible for antigen binding. Key Fc receptor binding regions of the structure are shaded in purple. **(B)** IgG allotype motifs. Allotypes are motifs identified by serological cross reactivity between donors. These motifs are mapped to the same alignment shown in Figure 2 for all IGHG alleles. Residues known to directly interact with Fc receptors are highlighted in blue (FcγRI) (81), red (FcγRIIa & FcγRIII) (82, 83), and green (FcRn) (84).

While allotypes have long been the defining feature of Ig isotype diversity we argue that the richer diversity found at the allelic level is likely to have profound consequences for our use and understanding of antibodies. These mutations have largely been ignored due to a lack of immunogenic phenotype; however, they likely play a crucial role in host immunity or in the mechanism of monoclonal antibody drugs (Figure 1A). This review summarizes the current known natural diversity of human immunoglobulin constant regions, the implications for antibody function beyond what is known for historically defined antibody allotypes, and gaps in our current knowledge of IGHC/IGLC allele function.

## The Challenges of IGHC/IGLC Allelic Discovery

The extent of IGHC/IGLC allelic diversity has been overlooked for many years due to the technical inaccessibility of the highly homologous IGH genomic locus. Approaches requiring the assembly of short DNA sequencing reads and genome-wide association studies (GWAS) have struggled to resolve individual genes in this locus (16, 17). These challenges have been compounded by the common practice of sampling circulating B cells that have undergone multiple instances of unique rearrangements, including class switch recombination, resulting in an IGH locus of inconsistent length and composition (17). It is particularly notable that GWAS gene probes for detection of single nucleotide polymorphisms (SNPs) in this region are largely absent from modern arrays and any identified SNPs have been difficult to assign to the correct immunoglobulin domain (18). The result has been a slow and very manual accumulation of known IGHC/IGLC allelic sequence data over more than 30 years.

**FIGURE 2:**
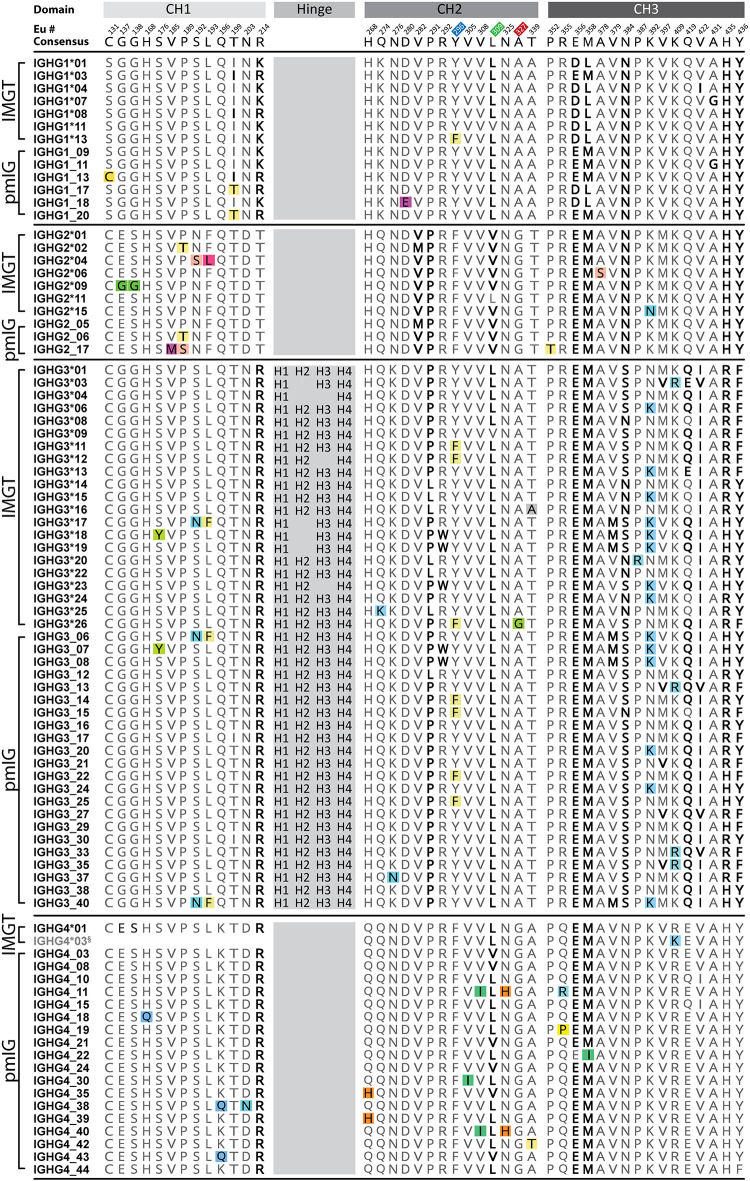
Unique amino acid mutations within IgG heavy chain alleles (IGHG) of all subclasses. IGHG (IgG1, IgG2, IgG3, IgG4) were obtained from IMGT and pmIG databases, aligned, and corresponding allotype or isoallotype motifs mapped as indicated in bold (3, 5, 20). Isoallotypes have only been reported in certain subclasses despite the often ubiquitous presence of these motifs. Residues are numbered according to the Eu numbering convention. Colored boxes represent unique amino acid differences between alleles that are not associated with known allotypes and for which the functional impact is largely unknown. Residues known to directly interact with Fc receptors are highlighted in blue (FcγRI) (81), red (FcγRIIa & FcγRIII) (82, 83), and green (FcRn) (84). H1, H2, H3, H4 indicate amino acid sequences for each of the hinge exons in IGHG3 alleles. ^§^Partial alleles where complete allele sequence information is missing.

Recently, specifically targeted PCR approaches and the curation of high-quality sequencing data, such as the 1,000 genomes project, have enhanced our ability to detect variation in this region at the genomic level (19, 20). In the past 2 years alone, more than 250 new IGHC/IGLC allelic variants have been discovered. Because a large proportion of the newly discovered diversity is protein coding and does not map to known allotypes (Figures 1B, 2), the consequences for antibody function are unknown. There is agreement with allotype population data in that IGHC allelic prevalence is heavily biased toward different ethnicities as evidenced by the differential distribution of alleles between five superpopulations; Africans, Americans, East Asians, Europeans, and South Asians (20). Within each allele, variation is largely confined to certain nucleotide positions suggesting the functional consequences of these alleles may be driving evolutionary selection over time. It is likely some of this variation was introduced via admixture from archaic hominins (21). Nonetheless, it remains evident that the true breadth of constant domain allelic diversity can only be uncovered once many more populations are investigated.

## Mechanisms by Which Constant Domain Diversity Influences Function

### Antibody Stability

Diversity in the constant domain is inherently linked to antibody function both *in vivo* and *ex vivo*. At a fundamental level, changes to the amino acid sequence can greatly influence both antibody assembly and stability. For example, the IgG4 subclass is well known to carry out Fab-arm exchange to create naturally bispecific antibodies which are thought to contribute to anti-inflammatory properties typical of IgG4 antibodies (22, 23). While most IgG4 alleles have arginine at position 409, IGHG4^∗^03 has the non-allotypic polymorphism, K409 (Figure 2). This mutation inhibits Fab-arm exchange by stabilizing CH3-CH3 interactions of the antibody (22, 24). Occurrence of this natural variant suggests there are benefits to remaining monospecific, however, these have yet to be explored in detail.

Other IgG subclasses (IgG1, IgG2, IgG3) also have allelic variants that influence CH3-CH3 interaction stability, despite not carrying out Fab-arm exchange. These include variations at positions 392 and 397 which are situated at the edges of the CH3 dimerization interface (24). Asparagine at position 392 significantly weakens CH3-CH3 interaction compared to K392 and the resulting IGHG3^∗^03 allele dissociates significantly faster than IgG4 molecules. It is proposed these point mutations influence antibody aggregation dynamics that may be important for the stability of mixed antibody populations in therapeutic formulations (25). Mutations found in the CH3 domains of other subclasses and isotypes may also impact antibody stability but have yet to be investigated.

### Fc Receptor Binding

A delicate balance of immune modulation is mediated by antibody interaction with Fc receptors. Disrupting or enhancing binding affinity for key activatory receptors [FcγRs (FcγRI, FcγRIIa, FcγRIIIa, TRIM21), FcαRs (FcαRI, Fcα/μR), FcεRs (FcεRI, FcεRII) and FcμRs (FcμR, Fcα/μR)] and inhibitory Fc receptors [FcγR (FcγRIIb)] can have significant consequence. These interactions are responsible for driving potent antibody-dependent cell-mediated actions, including cytotoxicity (ADCC), phagocytosis, trogocytosis, degranulation responses, antiviral responses, and cytokine release, all of which have been extensively reviewed elsewhere (26–28). As a general rule, the strength/pattern of binding to each receptor is correlated to the intensity of the immune response, with high affinity interactions directing stronger responses. Extensive mutational studies from antibody engineering have provided valuable insight into how Fc domain point mutations can alter antibody function (29–32). Not only can single heavy chain point mutations massively alter Fc receptor selectivity ratios (33), but these mutations do not need to be proximal to receptor binding sites (34).

Investigations into allotypes have long implicated a potential for perturbed interactions with Fc receptors. In particular, FcγRIII mediated NK cell cytotoxicity by clinical antibodies is diminished or enhanced by certain allotypes (35, 36). Very recently, a systematic study of Fc receptor binding to 27 unique IgG alleles found variation in the IgG3 class of antibodies contributed to altered Fc receptor binding (37). These effects were linked independently to both variation in the hinge length and to the presence of unique point mutations at positions 291, 292, and 296 in the CH2 domain. Differences in observed Fc receptor binding led to altered ADCC capacity thus highlighting the functional consequence of this diversity. Natural heavy chain variation is also proving to be beneficial for passive immunization strategies. A potent anti-HIV antibody response was linked to the expression of two IGHG3 alleles; IGHG3^∗^01m (GenBank:MK679684) and IGHG3^∗^17 (38). Both alleles carry polymorphisms (E419 and K392, respectively) that do not define allotypes but are implicated in improved binding to FcγRIII and FcγRIIb receptors and enhanced ADCC responses.

### FcRn Binding

Neonatal receptor binding (FcRn) is important for enhancing the circulating half-life of antibodies in serum, the transfer of passive immunity to neonates, and in the uptake of exogenous antibody bound antigens (39–41). Antibodies are recycled in a pH dependent manner with binding occurring at ∼pH 6.5 within pinocytosed endosomes and released when returned to pH 7.4 at the cell surface. Altering the binding affinity of the CH2/CH3 domain interface at either of these pHs can have immense consequence and has been well established for allotypic variants of clinical antibodies (42). In certain IgG3 alleles, a single amino acid change, R435, reduces binding to FcRn at low pH despite enhancing binding at neutral pH (43, 44). This results in out-competition by circulating IgG1 antibodies for FcRn binding sites and a greatly shortened half-life relative to other IgG subclasses.

### Classical C1q-Mediated Complement

Binding of C1q to IgG or IgM is the first step in the classical complement-dependent cytotoxicity (CDC) cascade that culminates with the formation of a membrane attack complex that lyse target cells (45). As with FcγR binding, C1q interacts with amino acids in the CH2 domain so variants at or near this interface can influence binding and thus CDC immune response. Although allotypic effects have been reported to affect C1q binding in other species (46), little effect has been reported in humans (47). Instead, alterations to the length of the hinge region in IgG3, particularly truncations, have been shown to enhance C1q activity (48). A wide range of allelic hinge diversity is found in IgG3, but it is currently unknown how this might influence C1q binding.

### Antigen Binding

Selective antigen recognition underpins both the evolutionary and clinical success of antibodies. A longstanding dogma of immunology postulates antigen binding is determined by the variable domains of the heavy and light chains while effector function and isotype are determined by the constant domains alone (49). However, there is growing evidence that this theory is oversimplified. Not only do B cell superantigens bind non-canonically to antibody domains (50), but some antigen binding domains influence effector function and constant domain selection can affect antigen binding (18, 49, 51). Abundant evidence from isotype switched antibodies possessing identical variable domains indicates point modifications to the CH1 domain can affect the conformation of the antigen binding pocket and subsequent affinity (52, 53). Several alleles (IGHG2^∗^02, ^∗^04, ^∗^09 and IGHG3^∗^17, ^∗^18) have amino acid substitutions in the CH1 domain, and diversity in light chain constant domains is well established, although to our knowledge the influence of these mutations on antigen binding have not been investigated. It is less intuitive to note mutation in the CH2/CH3 domains can also propagate allosteric changes to modulate antigen binding (54). Differences in antigen binding have been found for different alleles with the same variable region, although without high resolution analysis to determine exactly which mutations are contributing to the effect (55).

## Implications of Constant Domain Diversity in Disease

Immunoglobulin allotypes and haplotypes have long been associated with susceptibility to infections and diseases including breast cancer (56), autoimmunity (57, 58), malaria (39, 59), herpes (16, 60, 61), and hepatitis C (62, 63). More recently, studies have even linked constant domain diversity to longevity observed in certain populations (64). Often allotypic diversity is linked with serum abundance of antibodies targeting the infectious agent. For instance, antibody titers against Hepatitis C virus E1E2 glycoproteins are, in part, determined by G1m1, G1m17, G3m5, and G3m13 motifs (Figure 1B) (63) and are prognostic for faster recovery (65). Similarly, breast cancer patients carrying the G2m23 (56) or Km1 kappa allotypes (66) tend to have higher levels of IgG antibodies, which can be associated with better patient outcomes. The focus on allotypic motifs in these studies means the exact allelic context is uncertain and therefore which amino acids may be contributing to the observed phenotypes remains unknown.

In recent years, high quality genomic datasets have provided evidence implicating non-allotypic IGHC diversity in human disease. In line with the observations from allotypic analyses, a key consequence of variation is marked differences in antibody serum abundance. Jonsson et al., reported variant P189T (SNP:rs11627594) in the CH1 domain of IGHG2^∗^02 decreases global expression of IgG molecules (67). Importantly, their evidence also extended to isotypes other than IgG for which less diversity has been discovered to date. The study found a novel variant of IGHA1, also reported by Khatri et al. (encoding P85.1S; IMGT numbering, SNP:rs117775520), is associated with high IgA concentration. In Alzheimer’s disease, advances in the quality of exome sequencing have led to data reporting an association of three IGHG3 alleles with severity (68). One of these variants (Y296F) is non-allotypic and found in alleles IGHG3^∗^11, IGHG3^∗^12, IGHG3^∗^26. The underlying mechanism for this association is yet to be explored. Similarly, SNP associations have been reported in IGHG3^∗^18 (S176Y SNP:rs201430154) to increase the risk of inhibiting components of protein replacement therapies in hemophilia (69). The importance of considering non-allotypic allelic variation is further highlighted by the discovery of a SNP in the cytoplasmic tail of IgG1 which is prevalent in cases of systemic lupus erythematosus (57). This variant has major implications for the humoral response upon infection or vaccination, and in autoimmune disorders, by enhancing IgG1 expression. Beyond these studies, the role of constant domain diversity in disease remains broadly underexplored, especially in light of new allele discoveries, and despite a critical antibody role in adaptive immune defense.

## Potential Implications for Therapeutics and Diagnostics

Analysis of anti-drug antibodies has suggested that unique variable region motifs rather than allotypes have greater immunogenic potential (12, 70). Nonetheless genetic variation in antibody constant domains is considered critical to antibody design and most clinical antibodies are either of the G1m17 or G1m3 allotypes (11). We also note the emergence of therapeutic antibody formats entirely lacking constant domains e.g., Nanobodies or BiTEs (71). As IGH allelic discovery accelerates, the choice of therapeutic backbones will become even wider and allogenic potential should not be discounted. Additional consideration needs to be given to the influence of allelic selection on immune effector function especially considering the polymorphisms present in several of the Fc receptor genes (FcγRIIa-R131H, FcγRIII-F156V) (72). More studies are required to determine the influence of new allelic diversity on potential effector functions. The influence of diversity in the constant domain also impacts antibody serum persistence, especially when in competition with natural allelic variants that may have vastly different FcRn binding affinities.

Both monoclonal and polyclonal antibodies continue to be essential reagents for rapid diagnostic testing and as probes in research despite contributing to a “crisis of reproducibility” in the scientific literature (73). Constant domain diversity can introduce error in two primary ways. First, purification of these reagents from complex mixtures such as serum or culture media requires the use of chromatographic approaches. The commonly used Fc-binding protein, Protein A, can only bind to and purify certain allelic variants of IgG3 carrying H435 (44, 74). Missing certain antibody subclasses could impact the functional composition of certain antibody preparations. Second, detection blind spots are created as common secondary detection-antibodies have incomplete reactivity to all antibody alleles or may even possess unwanted cross-reactivity to other isotypes (75). A key example is the discovery of an FDA approved anti-Kell monoclonal that fails to detect IgG3^∗^03 and IgG3^∗^13 antibodies, a false-negative result that could lead to hemolytic complications in new-borns.

## Future Perspectives

The discovery of large numbers of new alleles in recent years exemplifies the idea that much of the diversity within the constant region of antibodies has been technically inaccessible and therefore largely underestimated. It is probable the full extent of diversity is yet to be realized, as suggested by comparative genomics studies in non-human species (76), especially for antibody isotypes other than IgG and for kappa/lambda light chains. High quality genomic assemblies such as the upcoming “All of Us” million genome project have the potential to provide rich haplotype information for the IgH region (77). We highlight the recent development of new genomics tools and databases specifically aligned with diversity in this region. Rodriguez et al., report the use of a novel bioinformatics tool, called IGenotyper, used in conjunction with IGH-targeted long-read sequencing to characterize variation in the IGH locus in eight samples (78). This technology can be readily multiplexed to enable population-scale analyses. Furthermore, a population matched IG (pmIG) database^1^ has been released which compiles known immunoglobulin allelic variants from the 1000 Genomes project.

Several questions are inevitably raised as new diversity is discovered. Key among these is how constant domain diversity influences the immune response with implications for identifying those at risk for disease, infection susceptibility, and in the design of more effective vaccine strategies. There is growing evidence that antibodies can interact with non-canonical Fc receptors (TRIM21, DC-SIGN) and display functions that are not typical of antibodies. This includes activities characteristic of proteins (enzymes, cytokines, or chaperones) and the use of atypical means to neutralize pathogens or regulate the immune system (79). The role of constant domain diversity has not yet been investigated for these instances. Likewise, the influence of IGLC diversity on antibody function will be important to explore in greater depth. Together we expect this information will enhance the design and delivery of next-generation antibody drugs and enable higher accuracy in diagnostic approaches.

## Author Contributions

WK conceived of the review topic. AW and WK planned and wrote the review. Both authors contributed to the article and approved the submitted version.

## Conflict of Interest

The authors declare that the research was conducted in the absence of any commercial or financial relationships that could be construed as a potential conflict of interest.

## Footnotes

1 https://github.com/InduKhatri/pmIG
